# Effectiveness of an integrated smoking cessation service model on smoking status: A preliminary study

**DOI:** 10.18332/tid/155375

**Published:** 2022-11-21

**Authors:** Kamollabhu Thanomsat, Jintana Yunibhand, Sunida Preechawong

**Affiliations:** 1 Faculty of Nursing, Chulalongkorn University, Bangkok, Thailand

**Keywords:** smoking cessation, smoking status, integrated

## Abstract

**INTRODUCTION:**

Smoking cessation has been considered a benefit for smokers. This study aimed to investigate the effect of an integrated smoking cessation service model (ISCSM) on enhancing cessation among smokers in a community setting.

**METHODS:**

The participants were 144 smokers allocated into two groups, experimental and control with 72 participants each. The ISCSM comprised two sessions: 1) smoking cessation service design and training smoking cessation capacity for the Community Health Workers (CHWs) by nurses; and 2) integrated smoking cessation service delivery. The CHWs offered brief advice for smoking cessation for smokers through home visits under supervision by nurses, then referred to proactive multisession intensive telephone counselling that was behavioral therapy with follow-up. In contrast, the control group received Thai therapy, which was mouthwash. The 7-day point prevalence abstinence (PPA) was assessed 30 days after the quit date. The probability of quitting between the experimental and control groups was calculated by the risk ratio (RR). Propensity score matching was performed to analyze the treatment effect after balancing the covariate factors.

**RESULTS:**

The probability of quitting smoking successfully among the participants in the experimental group was 7.5 times higher than the control group (χ^2^=46.18, RR=7.50, p<0.001). For the treatment effect tested by the PSM, the ISCSM efficiently impacted the 7-day PPA at 30 days among smokers in the experimental group after balancing the covariates (SS=0.281, MS=0.281, df=1, F=13.20, p<0.001).

**CONCLUSIONS:**

The findings of this study indicate that the ISCSM is an efficient, powerful intervention for enhancing smoking cessation.

## INTRODUCTION

Tobacco use is a significant risk of health problems worldwide, including in Thailand^1^. Furthermore, there have been numerous recent studies demonstrating that cigarette smoking is a significant risk factor for serious consequences in COVID-19 individuals^2^. Thailand’s smoking cessation service system has been implemented by the guideline of the World Health Organization Framework Convention on Tobacco Control (WHO-FCTC) Article 14^3^. The smoking cessation services provided by healthcare providers have been launched in various features, such as the Smart Quit Clinic, Thailand National Quitline (TNQ), etc. However, the number of smokers who can quit smoking successfully has been lower than the expectation of the third national strategic plan for tobacco control (2022–2027) indicator^4^, which shows that the prevalence among Thai smokers needs to decrease to 14% by the end of 2027. It is plausible that current smoking cessation methods have numerous flaws and obstacles. According to the assessment of the WHO-FCTC Implementation Thailand^5^, the Thai government put much effort into providing smoking cessation services by healthcare professionals; however, the previous smoking cessation service that Thailand healthcare providers offered for smokers was compartmentalization—the previous smoking cessation service system did not cooperate to increase the number of smokers quitting successfully. To reach Thailand’s national tobacco control indicator, these barriers must be addressed and the existing smoking cessation service systems in particular need to work together.

Concerning one of the WHO-FCTC Article 14 guidelines, the smoking cessation system should prioritize primary healthcare services beginning with brief interventions and referral to the cessation quitline service. Nurses have a critical role in detecting smokers, determining the most appropriate techniques for each smoker, and monitoring the expected outcomes of delivered care in the primary care context^6,7^. Furthermore, they usually accurately assess and appreciate the setting that facilitates smoking cessation. They have played an essential role as coordinators among the affiliated organizations. However, the best strategy for implementing smoking cessation in primary care is inconclusive, mainly providing smoking cessation services by coordinating with the existing tobacco cessation system such as the TNQ. Therefore, this study built up the new smoking cessation service model in the primary healthcare service to increase the abstinence rate among smokers and aimed to present the effectiveness of an integrated smoking cessation service model (ISCSM) on smoking status.

## METHODS

### Study design

This study was quasi-experimental involving two groups with a post-test only design. The participants in the experimental group participated in the ISCSM, while the participants in the control group were offered usual care (the control group received Thai therapy which was mouth wash). The 7-day PPA at 30, 90, and 180 days after the quit date, was assessed.

### Theoretical model of the intervention

The socio-ecological model, focusing on the linkage between a person’s environment and health problems, was used as a guideline to design the smoking cessation intervention, including individual, interpersonal, organizational, community, and public policy. There are various levels of development, including: The policy levels at the community level and the organization level comprised: development of the potential capacity of the CHWs in helping smokers quit through home visits, and referring smokers’ information for receiving intensive staged-based^8^ proactive telephone counseling for smoking cessation by the TNQ. Furthermore, nurses working at Thailand’s Health Promoting Hospitals (HPHs), which are hospitals in primary care settings, were trained to supervise CHWs according to the study protocol. Individual and interpersonal levels included providing brief advice to encourage smokers to quit through home visits, referring the smokers’ information to the TNQ counselors via the mobile application for intensive staged-based^8^ proactive telephone counseling for smoking cessation, and follow-up to prevent relapse. Furthermore, the transtheoretical model^9^ was used as the theoretical support for the outcome identification – smoking status.

### Participants

The sample size was calculated based on the power analysis and effect size determined by a former study^10^, with significance criterion at α=0.05, power at 0.80, and effect size at 0.3^11^. In all, 144 participants were allocated into the two groups, with 72 in each. The participants were smokers from four communities in Thailand’s Nakhon Pathom Province, located in the country’s central region. This was due to the fact that the smoking prevalence in this province^12^ was still more than the Thailand National Strategic Plan for Tobacco Control indicators. Furthermore, because it comprises urban and rural areas, this province could represent all provinces. There are seven districts in Nakhon Pathom, of which the Mueng district, consisting of urban and rural areas and having a high smoking prevalence of tobacco use, was chosen for this study because it is representative of the province and also exemplifies the current smoking problem. It is made up of 18 HPHs. A multi-stage random sampling strategy was used to select the HPHs, and two were picked at random to represent HPHs. Inclusion criteria included: 1) being a regular smoker – using cigarette and roll-your-own tobacco for at least two years; 2) no diagnosis of severe respiratory diseases such as chronic obstructive pulmonary disease, cancer, etc., mental health disorders, alcohol or substance abuse, and autism; 3) no diagnosis of hearing loss and learning disorder; 4) able to speak and read in Thai and willing to participate in the program; and 5) aged ≥18 years. Exclusion criteria were the participants did not complete the program and/or developed underlying diseases complications.

To avoid the threats of validity, the experiment and control group participants were divided into two groups using a matched-pairs design^13^ at the beginning. Firstly, the researcher recruited the smokers from the name list reported by the HPHs, then matched the indicated qualifications, including age, nicotine addiction level, stage of change, tobacco use duration, and type of tobacco use, that were similar in each pair. Then, within each pair, one subject was assigned to the experimental group, and another was assigned to the control group. Secondly, the treatment effect on the outcomes between the experimental and control groups was analyzed using propensity score matching (PSM).

### Procedure

We built up the major components of the smoking cessation program on the literature research and convened a conference with stakeholders, which included two sessions.

Researchers and affiliated organizations established smoking cessation services, and Community Health Workers (CHWs) – crucial healthcare volunteers in Thailand who have a responsibility to take care of people in the village – helped researchers build their smoking cessation capability. Furthermore, nurses operating in the HPHs were preparing to supervise CHWs following the study protocol. After completing the training program, the CHWs research assistants were examined on their knowledge of smoking cessation services and support behavior to ensure the intervention’s validity. The study protocol, study handbook, research instruments, smoking cessation helping video, tobacco use recording, brief advice, and equipment, were created. Five experts were then asked to approve the study tools for content validity. Following that, all instruments were updated in accordance with the experts’ suggestions.

The study protocol, information, and informed consent form were all ethically reviewed to ensure confidentiality and anonymity before the intervention.

**Figure 1:**
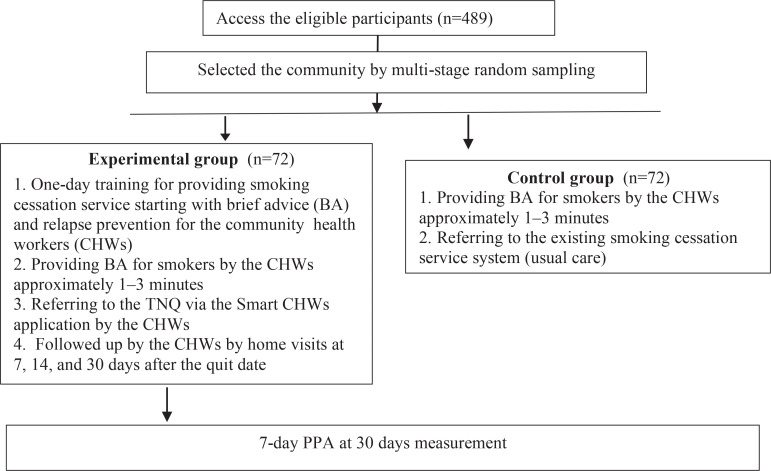
The study protocol

Researchers and nurses who work in the HPHs serve as supervisors to support the CHWs, while the CHWs serve as research assistants. To begin, the CHWs visited smokers in their homes and gave them brief counseling under supervision by nurses working in the HPH. The CHWs collected the data once the participant agreed to participate in the experiment. The smokers’ data were then sent to the TNQ, who used stage-match intervention to provide proactive multisession intensive telephone counseling^14^. The participants were offered only behavioral modification support. To prevent relapse, the TNQ and the CHWs then offered follow-up at 7, 14, 30 days after the quit date. Meanwhile, the control group received usual care, defined as smoking cessation services delivered by CHWs trained under the Quit for King program – the Thai government’s national smoking cessation project dedicated to King Rama IX, as reported by the HPHs. First, the smokers were offered brief advice for approximately 1–3 minutes to increase their intention to quit by the CHWs. Then the smokers were referred to the HPHs’ existing smoking cessation service system, including *Vernonia cinerea (L.) Less* oral spray, and 0.5% sodium nitrate mouth wash. After that, 7-day PPA at 30 days was measured by the CHWs. The study protocol is illustrated in Figure 1.

### Study measures

Participant data collected in the beginning included: 1) demographic data including gender, age, education level, occupation, underlying disease, type of tobacco, and smoking duration; 2) heavy smoking index (HSI)^15^; and 3) staging questionnaire^16^. Finally, the study participants 7-day PPA at 30, 90, and 180 days after the quit date was assessed by the CHWs. In this study, only the 7-day PPA at 30 days is analyzed.

### Statistical analysis

The data were analyzed using the statistical package for the social science for Windows (SPSS). For all analyses, a significant level of p<0.05 was utilized. Descriptive statistics were performed in the experimental and control groups to explain sociodemographic data, readiness to quit, nicotine addiction level, and smoking status. The sociodemographic data, readiness to quit, nicotine addiction level, and motivation to quit were used to assess the differences among the experimental and control groups using statistical tests based on the measurement scale. The proportion of smokers who quit after 30 days, as indicated by the 7-day PPA, was compared using descriptive statistics between the experimental and control group. The risk ratio (RR) was used to calculate the probability of abstinence rate in the experimental and control group. Moreover, the treatment effect on the outcomes between the experimental and control group was analyzed using PSM. In order to avoid the threat of validity, the covariates from the literature review include age^17^, the total number of years for education^8^, underlying disease^18^, smoking duration^19^, and nicotine addiction level (HSI score)^20^. All of them were determined as covariate factors when the treatment effect was calculated.

## RESULTS

The study participants were divided into 72 smokers in each group. The majority of the participants (93.1%) were male. More than two-fifths of the participants in the experimental group (43.1%) and more than half of the participants (55.6%) in the control group were in the age group 41–59 years (mean=41.8 years, SD=9.2; mean=42.6 years, SD=9.2, respectively). The majority of the participants (80.6% and 82.0%, respectively) graduated from secondary and higher education. Roughly half of the participants in both groups had no underlying disease (51.4% and 51.4%, respectively). Chi-squared test was used to assess differences in demographic characteristics at the pretest. The results revealed that the characteristics of the experimental and control group were not significantly different (Table 1).

**Table 1:** Personal demographic characteristics of the participants at baseline

*Characteristics*	*Experimental (n=72) n (%)*	*Control (n=72) n (%)*	*χ^2^*	*p*
**Gender**				
Male	67 (93.06)	67 (93.06)		1.00
Female	5 (6.94)	5 (6.94)		
**Age** (years)				
<41	39 (54.17)	30 (41.67)	2.32	0.31
41-59	31 (43.06)	40 (55.56)		
≥60	2 (2.78)	2 (2.78)		
Average age, mean ± SD	41.83 ± 9.15	42.65 ± 9.24		
Age range	27-65	26-63		
**Educational level**				
Primary and lower	14 (19.44)	13 (18.06)	0.05	0.83
Secondary and higher	58 (80.56)	58 (81.94)		
**Occupation**				
Labor	32 (44.44)	31 (43.06)	0.18	0.36
Agriculture	28 (38.89)	30 (41.67)		
Own business	9 (12.50)	7 (9.72)		
Other	3 (4.17)	4 (5.56)		
**Underlying disease**				
No	37 (51.39)	37 (51.39)		1.00
Yes	35 (48.61)	35 (48.61)		
Hypertension	25 (71.43)	27 (77.14)		
Diabetes mellitus	7 (20.00)	6 (17.14)		
Other	6 (17.14)	8 (22.86)		

Approximately half of the study participants in the experimental and control group used cigarettes (50.0% and 52.8%, respectively); 51.4% of the participants in the experimental group used tobacco for 21–30 years, while 45.8% of the participants in the control group used tobacco for 21–30 years (mean=24.4 years, SD=7.7; mean=24.0 years, SD=7.9, respectively); 97.2% of participants in both experimental and control groups had a nicotine addiction level in the moderate level (mean=3.1, SD=0.6; mean=3.1, SD=0.5, respectively). The chi-squared tested the difference in tobacco use patterns at the pretest. The results revealed that the tobacco use patterns of the experimental and control groups were not significantly different (Table 2).

**Table 2:** The tobacco use patterns of the participants at baseline

*Tobacco use patterns*	*Experimental (n=72) n (%)*	*Control (n=72) n (%)*	*χ^2^*	*p*
**Type of tobacco products**				
Cigarette	36 (50.00)	38 (52.78)	0.13	1.00
Roll-your-own tobacco	36 (50.00)	34 (48.22)		
**Smoking duration** (years)				
<10	1 (1.39)	1 (1.39)	5.79	0.12
11-20	16 (22.22)	28 (38.89)		
21-30	37 (51.39)	33 (45.83)		
≥31	18 (25.00)	10 (13.89)		
Average smoking duration, mean ± SD	24.36 ± 7.74	23.99 ± 7.93		
Range of smoking duration	10–50	10–48		
**Nicotine addiction level**				
Low	2 (2.78)	2 (2.78)		1.00
Moderate	70 (97.22)	70 (97.22)		
Average HSI score, mean ± SD	3.07 ± 0.57	3.13 ± 0.47		
Range of HSI score	1–4	1–4		

The risk ratio (RR) was used to calculate the probability of the smoking abstinence measured by the 7-day PPA at 30 days, and among smokers in the experimental group was found to be higher than the control group. The abstinence rate of study participants in the experimental group was 62.5%. Meanwhile, only 6 participants (8.3%) in the control group quit smoking, indicating that smokers in the experimental group had a 7.5 times higher probability of quitting smoking more successfully than in the control group (χ^2^=46.2, RR=7.5, p<0.001) (Table 3).

**Table 3:** The probability of quitting smoking between the experimental and control group, measured by 7-day PPA at 30 days

*Group*	*Quit n (%)*	*Relapse n (%)*	*χ^2^*	*RR (95% CI)*	*p*
Experimental (n=72)	45 (62.50)	27 (37.50)	46.18	7.50 (3.42-16.47)	<0.001
Control (n=72)	6 (8.33)	66 (91.60)			

RR: risk ratio. PPA: point prevelance abstinence.

Generally, quasi-experimental research is influenced by confounding variables and many covariate variables. This study determined the covariates from the literature review, including age^16^, the total number of years of education^8^, underlying disease^18^, smoking duration^19^, and nicotine addiction levels (HSI score)^20^. Thus, the propensity score matching (PSM) was performed to balance the covariates in the two groups and thus reduce the bias in the experimental research^21,22^. The finding demonstrated that the ISCSM affected the 7-day PPA at 30 days among smokers in the experimental group after balancing the covariates (SS=0.281, MS=0.281, df=1, F=13.20, p<0.001).

## DISCUSSION

According to the findings of this study, the ISCSM effectively boosted the abstinence rate among smokers. The CHWs might be considered as key human resources in Thailand’s basic healthcare system. They could give primary healthcare services to nearby neighbors. Furthermore, they are familiar with their client’s basic facts. Participants in this study’s experimental group were offered a smoking cessation service. This study supported the integrated smoking cessation service, which extends from primary care to the national smoking cessation service system. Smokers who got TNQ professional counselors – proactive multisession intense telephone counseling followed up by the TNQ and CHWs together – had a greater cessation rate than those who received standard care.

Furthermore, smokers who participated in the ISCSM had a greater abstinence rate than those who simply received TNQ smoking cessation services – 38.7% of smokers who had 7-day PPA^23^. It was consistent with a previous study^24^ that smokers who were offered counselling by village health workers were more likely to quit smoking than those who received standard treatment. The integration model is thought to improve ongoing smoking cessation. Furthermore, it was consistent with a previous study^25^ that coupled smoking cessation with a quick intervention followed by intense counseling by properly qualified specialists. In other words, the Thai government made an additional effort to provide smoking cessation services, such as using *Vernonia cinerea* ^26,27^.

The findings of this study have implications for nursing practice, nursing education, and national health policy. The results show that the seven-day PPA at 30 days after the quit date in the experimental group was higher than in the control group after completing the program. Therefore, nurses working in the primary care setting should utilize the components of this program to provide smoking cessation services. Consequently, they need to provide the smoking cessation assistance capacity for the CHWs or other healthcare volunteers and supervise them in offering smoking delivery in the community. Moreover, nurses working in other fields should cooperate with the community nurses working in the primary care setting to provide thorough smoking cessation for smokers. Based on the results of this study, in the nursing curriculum should be added a training program for smoking cessation assistance behavior for CHWs, especially community nursing subjects, and advanced nurse practitioners working in primary care units. In this study, the duration of the integrated smoking cessation service model in the primary care service system was 30 days; thus, further research should extend to a longer duration in order to achieve successful continuous abstinence of at least six months.

### Limitations

The study has some limitations. The results are only preliminary, and only for abstinence at 30 days. It cannot be concluded that smokers participating in this study could quit permanently. Some smokers use a combination of different types of tobacco. Furthermore, self-reported tobacco abstinence was not biochemically verified at follow-up. As a result, both experimental and control group outcomes might be influenced.

## CONCLUSIONS

According to the findings of this study, community nurses play an important role in providing smoking cessation services. Furthermore, community health workers (CHWs) are critical human resources in Thailand’s primary healthcare system. As a result, this study advocated a collaborative paradigm among community nurses, CHWs, and existing smoking cessation services, particularly the TNQ, in accordance with WHO-FCTC recommendations. The major finding of this study was that the abstinence rate among smokers in the experimental group was higher than that in the smokers in the control group, 30 days after quitting. As a result, there are important implications for nursing practice, education, and future research. First, nurses in primary care should use the components of this approach to deliver smoking cessation treatments. Second, a nursing curriculum for smoking cessation aid practice for CHWs, particularly community nursing subjects and advanced nurse practitioners working in primary care units, should be incorporated to the training program. Finally, a as only a 30-day integrated smoking cessation service model was implemented in the primary care service system, more research needs to be done over a longer period in order to achieve effective 6-month abstinence.

## Data Availability

The data supporting this research are available from the authors on reasonable request.
